# Artemisinin relieves osteoarthritis by activating mitochondrial autophagy through reducing *TNFSF11* expression and inhibiting PI3K/AKT/mTOR signaling in cartilage

**DOI:** 10.1186/s11658-022-00365-1

**Published:** 2022-07-28

**Authors:** Jin Li, Mengqing Jiang, Zhentang Yu, Chenwei Xiong, Jieen Pan, Zhenhai Cai, Nanwei Xu, Xindie Zhou, Yong Huang, Zhicheng Yang

**Affiliations:** 1 Department of Orthopedic Surgery, The Second Affiliated Hospital of Jiaxing University, Jiaxing, 314000 China; 2 Department of Pharmacy, The Second Affiliated Hospital of Jiaxing University, Jiaxing, 314000 China; 3 Department of Orthopedics, The Affiliated Changzhou No.2 People’s Hospital of Nanjing Medical University, Changzhou, 213000 China

**Keywords:** Artemisinin, PI3K/AKT signaling pathway, Autophagy, TNFSF11, Mitochondria, Osteoarthritis

## Abstract

Osteoarthritis (OA) is a widespread chronic degenerative joint disease characterized by the degeneration of articular cartilage or inflamed joints. Our findings indicated that treatment with artemisinin (AT) downregulates the protein levels of MMP3, MMP13, and ADAMTS5, which are cartilage degradation-related proteins in OA, and inhibits the expression of inflammatory factors in interleukin-1β (IL-1β)-stimulated chondrocytes. However, the mechanism of the role of AT in OA remains unclear. Here, we performed gene sequencing and bioinformatics analysis in control, OA, and OA + AT groups to demonstrate that several mRNA candidates were enriched in the PI3K/AKT/mTOR signaling pathway, and *TNFSF11* was significantly downregulated after AT treatment. *TNFSF11* was downregulated in the OA + AT group, whereas it was upregulated in rat OA tissues and OA chondrocytes. Therefore, we confirmed that *TNFSF11* was the target gene of AT. In addition, our study revealed that AT relieved cartilage degradation and defection by activating mitochondrial autophagy via inhibiting the PI3K/AKT/mTOR signaling pathway in IL-1β-induced chondrocytes. Furthermore, an OA model was established in rats with medial meniscus destabilization. Injecting AT into the knee joints of OA rat alleviated surgical resection-induced cartilage destruction. Thus, these findings revealed that AT relieves OA by activating mitochondrial autophagy by reducing *TNFSF11* expression and inhibiting PI3K/AKT/mTOR signaling.

## Introduction

Osteoarthritis (OA), also known as degenerative arthritis, is a chronic degenerative disease. During disease progression, all compartments of the joints undergo structural, functional, and metabolic changes involved in progressive articular cartilage degeneration, synovitis, and subchondral bone damage [1–4]. The pathological changes are irreversible, resulting in physical disability and affecting patients’ quality of life; overall, more than 230 million individuals are estimated to be affected by OA worldwide [5]. The pathogenesis of OA is related to joint injury, obesity, aging, heredity, and inflammation, although it is not fully understood. There are no effective strategies to cure OA completely, and the common clinical treatments include pain suppression [6]. Chondrocytes play an essential role in regulating cartilage and extracellular matrix homeostasis. Further, these chondrocytes are responsible for the biochemical synthesis and secretion of the extracellular matrix [7, 8]. Osteoarthritic cartilages are associated with chondrocyte hypertrophy, inflammatory processes, and chondrocyte death. Thus, understanding the pathological mechanisms of chondrocytes will help develop novel therapeutic measures for OA treatment.

Artemisinin (AT) is a widely known antimalarial drug [9]. In recent years, AT has received widespread attention because of its pharmacological properties and extensive pharmacological effects, which have been explored extensively. AT has been reported to be used as an anticancer drug by inducing lysosomal degradation of ferritin, improving free iron levels, and inducing cells to become more sensitive to ferroptosis [10]. AT has been reported to alleviate OA by regulating osteoclast formation and alleviating interleukin‑1β‑induced inflammatory response or apoptosis by regulating NF-κB signaling [11]. In addition, a recent study also demonstrated that AT played an essential role in anti-inflammatory and anti-apoptotic effects on IL-1β-induced chondrocyte-like ATDC5 cells [12]. However, our study showed that AT alleviates OA by regulating mitochondrial autophagy.

The *TNFSF11* gene, which encodes RANKL, is expressed in many different cell types and is responsible for osteoclast differentiation [13, 14]. The most crucial function of RANKL in osteocytes is regulating osteoprotegerin (OPG) release onto the bone surface. The OPG on the surface of osteocytes binds to RANKL on the surface of osteoblasts, thereby inhibiting osteoclastogenesis [15]. In addition, RANKL plays a vital role in the development of lymph nodes, final stages of mammary gland development, and optimal production of mature B and T lymphocytes [16, 17]. Recent evidence shows that RANKL may participate in subchondral bone alterations in OA. RANKL is secreted by a disintegrin and metalloprotease domain (ADAM) and MMP-7 [18]. In cartilage, decreased OPG:RANKL ratio induces chondrocyte apoptosis and matrix loss by degrading matrix metalloproteinases, such as MMP-13 or MMP-9 [19]. Moreover, changes in OPG/RANKL expression could lead to a pro-inflammatory phenotype in osteoarthritic osteoblasts [20, 21]. In human OA subchondral bone, RANKL could be regulated to influence the bone remodeling process. However, the mechanism of RANKL in regulating OA pathology remains unclear.

Autophagy is an intracellular degradation system that regulates energy metabolism, degrades impaired proteins, and maintains body homeostasis; it has been shown to restore the function of damaged chondrocytes, alleviating OA progression by protecting articular cartilage [22–24]. Mitochondria are the organelle sites for tricarboxylic acid cycle (TCA) progression and supply the electron transport chain (ETC) to manage energy production and cell distribution [25]. Thus, mitochondria and autophagy have a bidirectional regulation. Activation of autophagy degrades impaired mitochondria or other damaged organelles, whereas mitochondria are responsible for pro-survival functions of autophagy. For example, ischemic preconditioning in the heart causes a decrease in mitochondrial ATP production, which results in autophagy activation aggravating organ damage [26, 27]. Impaired mitochondria cause a decrease in the ATP:AMP ratio and induce autophagy by activating AMP-activated protein kinase (AMPK) or regulating mTOR signaling [28]. Mitochondria are frequently found engulfed in autophagosomes. In addition, the PI3K/AKT/mTOR pathway is widely explored as a fundamental intracellular signaling pathway that plays a vital role in many cellular processes, especially in cancer and metabolism disorders [29–31]. PI3K/AKT/mTOR signaling pathway could inhibit autophagy activation.

This study explored whether AT treatment alleviates IL-1β-induced chondrocyte production in vivo and in vitro. Our data showed that AT treatment suppressed PI3K–AKT–mTOR signaling, and *TNFSF11* was the most downregulated gene upon AT treatment in OA chondrocytes. A novel mechanism of AT treatment in OA was explored to show that AT activates autophagy by reducing *TNFSF11* and inhibiting PI3K/AKT/mTOR signaling in cartilage.

## Materials and methods

### Materials

AT was purchased from MedChemExpress (MCE, China), and dissolved in DMSO (dimethyl sulfoxide; Sigma). Then, the solution was added to the cell culture medium at various concentrations. MYH1485 was purchased from MCE, dissolved in DMSO (Sigma), and then added to the cell culture medium. Similarly, IL-1β was purchased from MCE, dissolved in DMSO (Sigma), and added to the cell culture medium. All these solutions were stored at −20 °C.

### Primary chondrocyte isolation and culture

Primary chondrocytes were extracted from the knee joints of five 6-week-old normal Sprague Dawley rats. All experiments were executed following animal approved protocols and guidelines. Cartilage was separated from the joints and washed with 1× phosphate-buffered saline (PBS) three times. After cutting the cartilage into small pieces, 0.25% trypsin was added for 30 min, followed by incubation with 2 mg/mL collagenase for another 4 h at 37 °C. The cells were then centrifuged at 1000 rpm for 5 min and cultured in Dulbecco’s modified Eagle’s medium (DMEM) (HyClone, USA) supplemented with 10% fetal bovine serum (FBS) (Gibco, USA) and 1% penicillin/streptomycin (HyClone, USA) in 5% CO_2_ in a humidified atmosphere in an incubator at 37 °C. The culture medium was replaced every 2 days. The chondrocytes in the third and fourth passages were used for further experiments.

### Induction of chondrogenesis in chondrocytes

Three groups were evaluated to explore the AT effects on IL-1β-induced chondrocytes: the normal group, the IL-1β group, and the IL-1β + AT group. The chondrocytes were preincubated with IL-1β (10 ng/mL) for 48 h and incubated with AT at different concentrations for 48 h. To study the association of AT with the PI3K/AKT/mTOR signaling pathway, MYH1485, an inhibitor of the PI3K/AKT/mTOR signaling pathway, was used.

### Transfection

pcDNA3.1 and pcDNA3.1-*TNFSF11* were purchased from JIMA GENE (China). Primary chondrocytes were seeded in six-well plates with a complete culture medium supplemented with 10 ng/mL IL-1β for 24 h. After primary chondrocytes reached 80% density, 4 μg/mL pcDNA3.1 and 4 μg/mL pcDNA3.1-*TNFSF11* were mixed with LipofectamineTM 2000 (Thermo Fisher Scientific) and incubated for 20 min at room temperature. Subsequently, 1.5 mL fresh medium was added to each well, and the mixture was added to the cells followed by incubation for 24 h. Western blotting was performed to determine overexpression efficiency.

### Cell counting kit-8 assay

Primary chondrocytes were seeded in 96-well plates with a complete culture medium supplemented with 10 ng/mL IL-1β at a density of 1 × 10^4^ per well for 24 h. The primary chondrocytes were then treated with 0, 2.5, 10, or 20 μM AT for 0, 24, 48, and 72 h. Subsequently, the cells in each well were incubated with 10 µL CCK-8 reagent (Sigma–Aldrich) for 1–2 h, and the optical density (OD) was measured at 450 nm using a microplate reader.

### Immunofluorescence analysis

Primary chondrocytes were cultured in glass slides with the complete culture medium supplemented with 10 ng/mL IL-1β for 24 h and then incubated with AT for 48 h. Following this, the cells were washed with 1× PBS and fixed in 4% paraformaldehyde for 20 min at room temperature. Fixed cells were permeabilized with 0.1% Triton X-100 for 10 min and blocked with 5% BSA for 1 h. Subsequently, primary antibodies against MMP-3 (Abcam, 1:100), beclin-1 (Abcam, 1:100), and *TNFSF11* (Abcam, 1:100) with 3% BSA were used to treat cells at 4 °C overnight. Following this, the cells were washed with 1× PBS three times, and incubated with secondary Alexa Fluor 594-conjugated antibody (Abcam,1:200) and Molecular Probes Alexa Fluor 488 phalloidin (Sigma–Aldrich) for 1 h at room temperature. The cell nuclei were then stained with 4′,6-diamidino-2-phenylindole dihydrochloride (DAPI; Sigma–Aldrich) for 5 min, coverslips were mounted on glass slides, and cells were imaged using a fluorescence microscope.

### Histology (H&E and Safranin O-Fast green staining) and immunohistochemistry

Knee joint tissues were fixed in 4% paraformaldehyde for 2 days and decalcified in 10% formic acid for 10 days before sectioning. Subsequently, bone tissues were dehydrated with graded ethanol, and embedded in paraffin. Following this, 3-μm-thick slices were cut and stained with H&E and Safranin O-Fast green. For H&E and Safranin O staining, the bone tissues were successively stained with hematoxylin (Sigma-Aldrich) and eosin (Sigma-Aldrich) for 5 min each. O-Fast green staining was performed according to the manufacturer’s instructions (Sigma-Aldrich). For immunohistochemical analysis, the bone tissues were fixed in 4% paraformaldehyde for 20 min and heated for 30 min for antigen retrieval. Following this, the bone tissues were treated with 3% H_2_O_2_ for 20 min and blocked with 5% BSA for 1 h at room temperature. Primary antibodies against ATG5 (Abcam, 1:300), MMP3 (Abcam, 1:300), and *TNFSF11* (Abcam, 1:200) were applied in 5% BSA at 4 °C overnight. Standard DAB staining was performed for chromogenic detection during immunohistochemical analysis. Bone tissues were photographed and observed using a Canon microscopic imaging system (model EOS-350D, Canon, Tokyo, Japan).

### Quantitative reverse-transcription PCR (qRT-PCR)

Primary chondrocytes were cultured in six-well plates at a density of 4 × 10^3^ and incubated with IL-1β for 24 h. The cells were then incubated with different AT concentrations (5 μM, 10 μM, 20 μM) for 48 h. Total RNA was extracted from isolated chondrocytes using TRIzol reagent (Invitrogen, Carlsbad, CA, USA) according to the manufacturer’s protocol. Equal amounts of RNA were quantified via cDNA synthesis using a reverse transcription kit (Applied Biosystems, Foster City, CA, USA). Real-time quantitative PCR was performed using the SYBR Green PCR MasterMix (Applied Biosystems, A25780), according to the manufacturer’s protocol. The expression of the GAPDH gene was used to normalize other genes, and the 2^−ΔΔCt^ method was used to analyze the relative expression of each gene. The primer sequences are listed in Table 1.

**Table1:** qPCR primer sequences for target gene

Primers	Forward (5′–3′)	Reverse (5′–3′)
TNF-α	GTCGTAGCAAACCACCAAGC	TGTGGGTGAGGAGCACATAG
IL-6	ATTGTATGAACAGCGATGATGCAC	CCAGGTAGAAACGGAACTCCAGA
IL-1β	GCAATGGTCGGGACATAGTT	GCAATGGTCGGGACATAGTT
ADAMTS5	CCCAAATACGCAGGTGTCCT	ACACACGGAGTTGCTGTAGG
MMP-3	TTTGGCCGTCTCTTCCATCC	GGAGGCCCAGAGTGTGAATG
MMP-13	GGACTCACTGTTGGTCCCTG	GGATTCCCGCAAGAGTCACA
TNFSF11	ACGCAGATTTGCAGGACTCGAC	TTCGTGCTCCCTCCTTTCATC
PTHLH	ACACCAAAAACCACCCTGTGCGGT	GAATCCTGTAACGTGTCTTGG
ATG-5	TGGACCATCAACCGGAAACT	CAAGGGTATGCAGCTGTCCA
ATG-7	GAGAGCCGATGGCTTCCTAC	CAGGTCAGCAGGTGCTACAA
Beclin-1	TTCAAGATCCTGGACCGAGTGAC	AGACACCATCCTGGCGAGTTTC
LC-3	ATCATCGAGCGCTACAAGGGTGA	GGATGATCTTGACCAACTCGCTCAT
GAPDH	GGCACAGTCAAGGCTGAGAATG	ATGGTGGTGAAGACGCCAGTA

### Western blot

Isolated chondrocytes were cultured in six-well plates at a density of 4 × 10^3^ and incubated with IL-1β for 24 h in the presence or absence of AT (5 μm, 10 μm, and 20 μm) for 48 h. Total protein was extracted from chondrocytes using a whole-cell lysis buffer and quantified via BCA protein assay. Protein samples were separated by SDS polyacrylamide gel electrophoresis (SDS–PAGE) for 120 min and transferred onto PVDF membranes. Subsequently, the membranes were blocked with 5% milk for 1 h at room temperature and incubated with primary antibodies at 4 °C overnight. Antibodies for MMP-3, MMP-13, ADAMTS5, LC3, beclin-1, ATG-5, ATG-7, PI3K, p-PI3K, AKT, p-AKT, mTOR, p-mTOR, IL-1β, IL-6, TNF-α, *TNFSF11*, and GAPDH were used, which were purchased from Abcam. The membranes were washed with TBST three times and incubated with secondary antibodies (1:5000) for 1 h at room temperature. The protein bands were developed using ECL and then imaged and quantified on a computer.

### Differential gene expression and gene set enrichment analysis

Total RNA was extracted from chondrocytes that underwent different treatments (A: control group, B: OA group, and C: OA + AT group) using TRIzol (Invitrogen, Carlsbad, CA, USA) for RNA sequencing (RNA-seq) and was analyzed using Illumina HiSeq platforms. Analysis of differential gene expression was performed using R package DESeq2. The fold change (≥ 2.0, corrected *P*-value ≤ 0.05) was set for upregulated and downregulated gene thresholds. Differentially expressed genes (DEGs) that showed statistical significance were filtered through a scatter plot. We identified DEGs between untreated cells (group A) and IL-1β-treated cells (group B), and DEGs between AT + IL-1β-treated cells (group C) and IL-1β-treated cells (group B). The Venn diagram showed an overlap between DEGs that were significant in AT + IL-1β-treated samples and those that were significant in IL-1β-treated samples; and the IL-1β-treated samples and untreated samples. The R package “clusterProfiler” was used to reveal the functions of the DEGs.

### Micro-computed tomography scanning

The left distal femurs of rats were extracted and fixed in 4% paraformaldehyde for 2 days, and scanned with a Scanco Viva CT40 micro-computed tomography (mCT) instrument. Parameters were set to 65 kV, 385 mA, 10 mm voxel size, and 300 ms exposure for the scans. Right femurs were fixed in 4% paraformaldehyde for 2 days and then decalcified with 10% EDTA for at least 10 days. The paraffin-embedded bone was sectioned for H&E staining and immunohistochemical analysis.

### Animal experiments

The destabilized medial meniscus (DMM) model was employed to mimic OA [4, 32]. In brief, 15 4-week-old male Sprague Dawley rats (200–250 g) were used, and the medial menisci of these were carefully resected without cartilage and ligament injuries. The rats were randomly divided into three groups: control group (no surgery; normal saline treatment, with injection time same as that in the OA group; ten knee joints from five rats, *n* = 10), OA group (surgery; normal saline treatment on the first day of every week from the 5th to the 8th week after surgery); and OA + AT group (surgery; 100 μL normal saline with 20 μm AT, with the injection time same as that in the OA group; five rats, *n* = 10). After sacrifice by an overdose of anesthesia, knee samples were extracted and fixed with 4% paraformaldehyde for 2 days, and then scanned via micro-computed tomography. The study was performed according to National Institutes of Health (NIH) guidelines (NIH Pub. No. 85–23, revised 1996) and the Laboratory Animal Management Regulations in China and adhered to the Guide for the Care and Use of Laboratory Animals published by the National Institutes of Health (2011), and the protocol was approved by the Ethics Committee of the Affiliated Changzhou No. 2 People’s Hospital of Nanjing Medical University ([2017]KY008-01).

### Data analysis

The Kyoto Encyclopedia of Genes and Genomes (KEGG; http://www.genome.jp/kegg/kegg1.html) and gene set enrichment analysis (GSEA, https://www.gsea-msigdb.org/gsea/index.jsp) were used to select influenced pathways. The working model in this paper was prepared from Figdraw (www.figdraw.com).

### Statistical analysis

GraphPad Prism 7.0 (GraphPad Software, CA, USA) was used to analyze the data, and the significance between the two groups was analyzed via Student’s *t*-test and Mann–Whitney test. Significance between multiple groups was analyzed via one-way analysis of variance (ANOVA). Quantitative data are presented as mean ± standard deviation (SD). *P*-value < 0.05 was deemed statistically significant (*P < 0.05; **P < 0.01; ***P < 0.001).

## Results

### AT reduced the expression of inflammatory factors and aggravated cartilage degradation and defection in OA chondrocytes

**Fig. 1:**
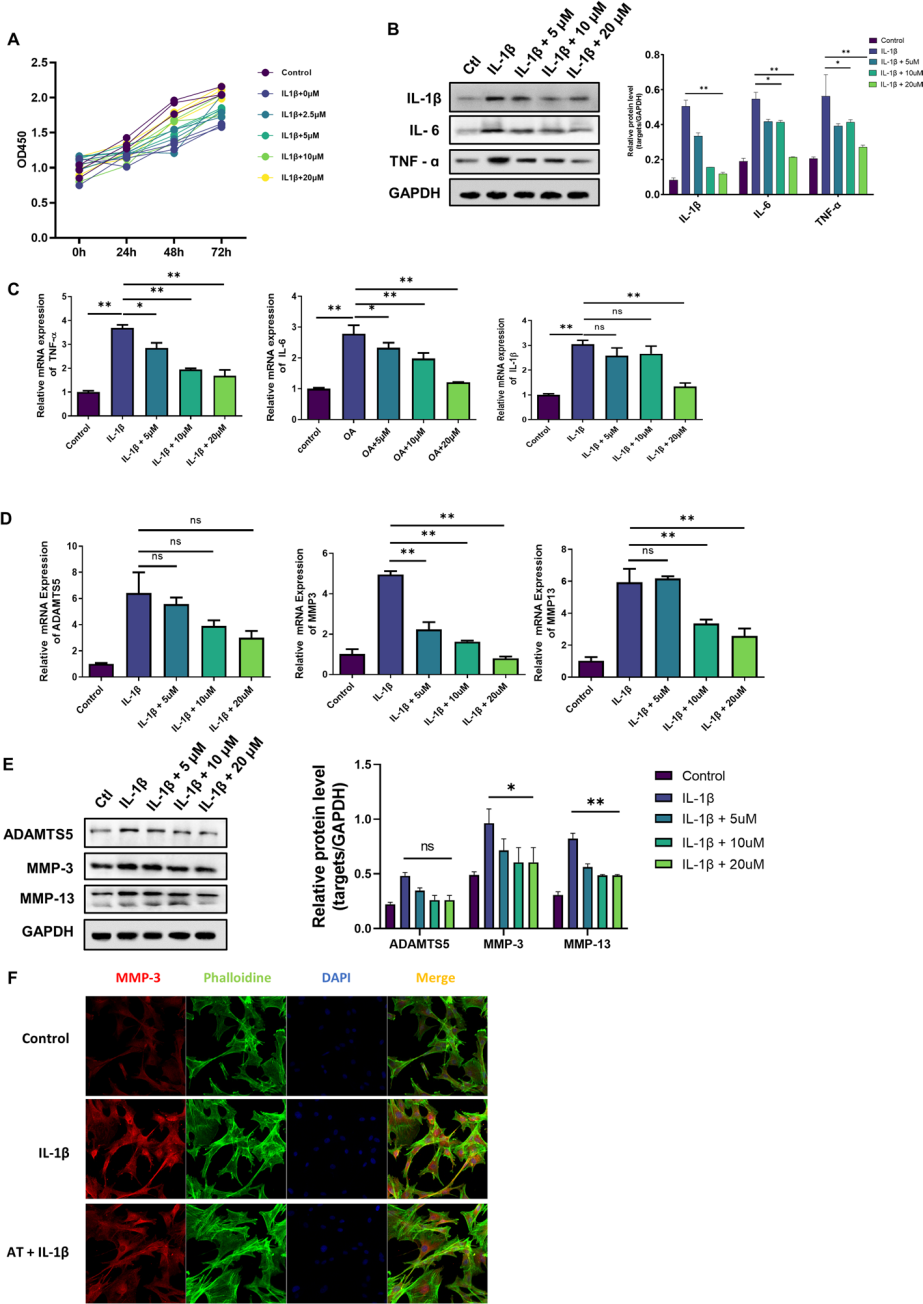
Chondroprotective and anti-inflammatory effects of AT on IL-1β-induced chondrocytes in vitro. **A** CCK8 assay was used to detect the optimum concentration of AT and the effect of AT in IL-1β-induced chondrocyte proliferation. **B** and **C** Western blot and real-time RT-PCR were performed to determine the expression of inflammatory factors, including IL-1β, IL-6, and TNF-α. **D** and **E** Real-time RT-PCR and western blot were performed to determine the expression of ADAMTS5, MMP-3, and MMP-13, which are cartilage matrix-related proteins. **F** Immunofluorescence staining for MMP-3, phalloidin was stained for cellular actin, and DAPI was stained for nuclear. Control: without IL-1β; IL-1β group: with 10 ng/mL; IL-1β + AT group: with 10 ng/mL IL-1β and 20 μM AT. All experiments were performed in triplicate. Data represent mean ± SD (*n* = 3), *P < 0.05, **P < 0.01, ***P < 0.001

Different concentrations of AT (0, 2.5, 5, 10, or 20 μM) were added to chondrocytes for 0, 24, 48, and 72 h with IL-1β to determine whether AT affects normal and OA cartilages. First, CCK8 was used to determine the influence of AT on OA chondrocyte proliferation. As shown in Fig. 1A, AT at concentrations of 0–10 μM slightly increased the proliferation of OA chondrocytes, but at a concentration of 20 μM, it significantly enhanced the proliferation of chondrocytes. Therefore, 20 μM was selected as the optimum concentration. Next, to study the anti-inflammatory and chondroprotective effects of AT on IL-1β-induced chondrocytes, chondrocytes were induced with IL-1β in the presence of various concentrations of AT (0, 5, or 10 μM). Inflammatory factors were dramatically inhibited by AT treatment in a dose-dependent manner (Fig. 1B, C). Levels of cartilage matrix-related MMP3, MMP13, and ADAMTS5 in OA chondrocytes were determined by qRT-PCR and western blotting. As shown in Fig. 1D and E, AT reduced the expression of MMP3 and MMP13 mRNA and protein levels. However, AT did not influence the ADAMTS5 mRNA level. We also detected the expression of MMP3 using a confocal microscope. As shown in Fig. 1F, the red fluorescence intensity in the AT + IL-1β group was weak compared with that in the IL-1β group. Based on the essential role of MMPs and ADAMTS5 in cartilage degradation, destruction of extracellular matrix (ECM) homeostasis is a key event in OA pathogenesis. The above-mentioned results indicated that AT protected chondrocytes from inflammatory degradation by reversing the upregulation of MMP3, MMP13, and ADAMTS5 induced by IL-1β stimulation.

### Identification of differentially expressed genes and gene set enrichment analysis

**Fig. 2:**
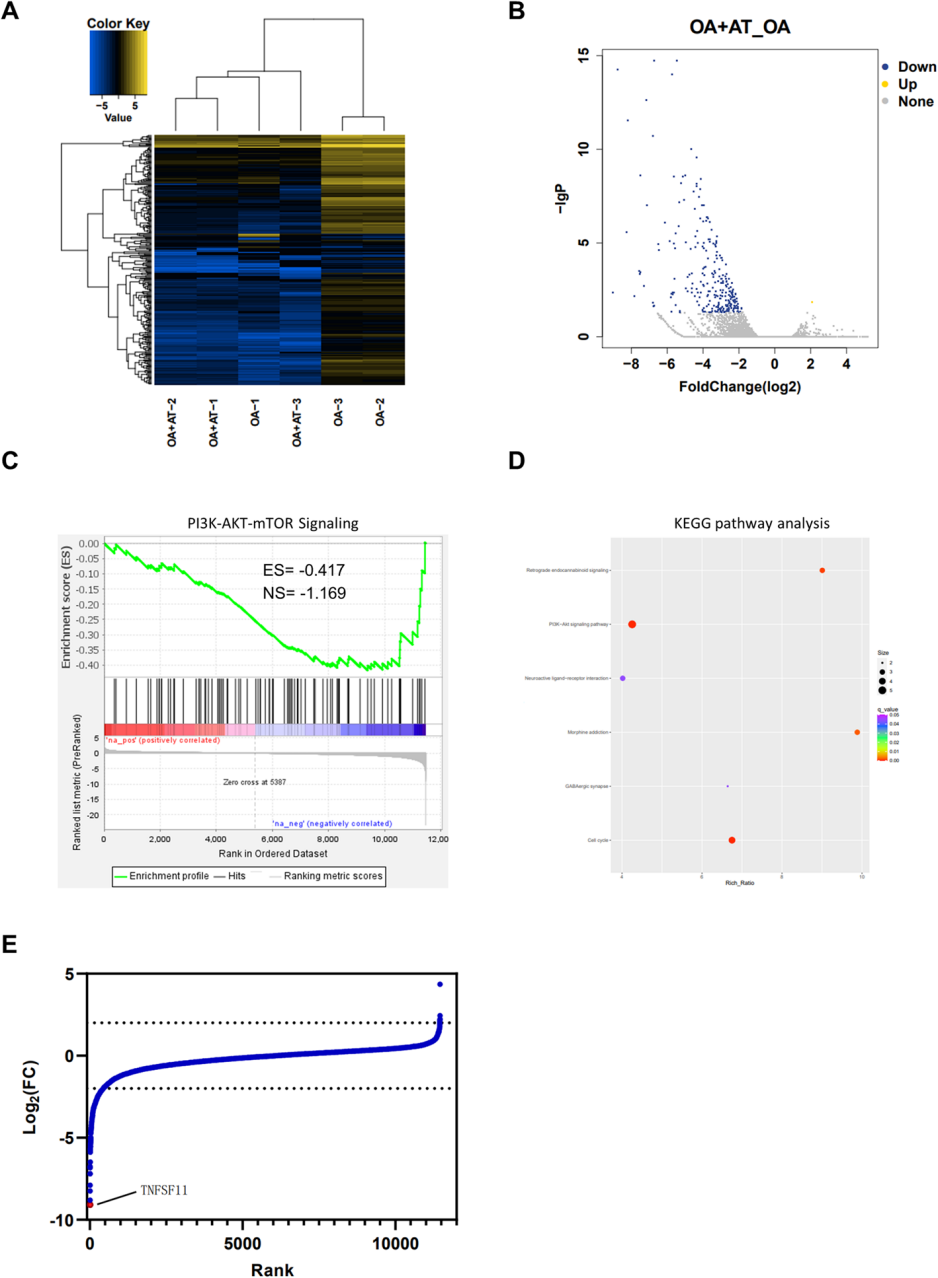
Transcriptomic and gene enrichment analysis upon AT treatment in OA chondrocyte cells. **A** Heatmap of expression of transcripts from triplicate experiments of OA and OA + AT. **B** Volcano plot of differential expressed genes. Yellow points represent upregulated genes for log_2_FC > 2, and blue points represent upregulated genes for log_2_FC < -2. **C** GSEA analysis of negative regulated genes shows PI3K–AKT–mTOR signaling. **D** The analysis of KEGG pathway enrichment for negative regulated genes. P13K-AKT-mTOR signaling pathway was enriched from these negative regulated genes. **E** Waterfall plot of fold change in gene expression in OA chondrocyte cells treated with 0.05 µM AT for 6 h; selected *TNFSF11* gene is highlighted

To investigate the mechanism underlying the effect of AT on OA, we performed RNA sequencing after AT treatment in OA chondrocytes. RNA sequencing and bioinformatics analysis were performed in two groups of chondrocytes, including (A) IL-1β group and (B) IL-1β + AT (20 μM) groups. All experiments in all groups were performed in triplicate. We first analyzed global gene expression in AT treatment and control groups, and the result indicated that most genes were downregulated upon AT treatment (Fig. 2A).

To identify which cellular pathway contributes to OA upon AT treatment, mRNAs with at least a 2.0-fold change in expression level and *P*-value ≤ 0.05 were selected (Fig. 2B). We then analyzed the gene enrichment of negative regulated genes via GSEA and KEGG, and the result revealed that AT treatment inhibited the PI3K–AKT–mTOR pathway in osteoarthritic chondrocytes (Fig. 2C, D). Moreover, to investigate which gene was the most downregulated by AT treatment, fold change (FC) in gene expression was evaluated between AT treatment group and the control group, and the results showed that *TNFSF11* was the most downregulated gene upon AT treatment (Fig. 2E). More importantly, *TNFSF11* was reported to activate the PI3K–AKT pathway by recruiting PI3K or AKT into their signaling complex, indicating that AT treatment downregulates the gene expression of *TNFSF11*, and downregulated *TNFSF11*, in turn, suppresses the PI3K–AKT–mTOR pathway [33]. In summary, our RNA-seq data identified that AT treatment suppressed the PI3K–AKT–mTOR pathway and effectively downregulated the gene expression of *TNFSF11*.

### *TNFSF11* was upregulated in OA and downregulated after AT treatment

**Fig. 3:**
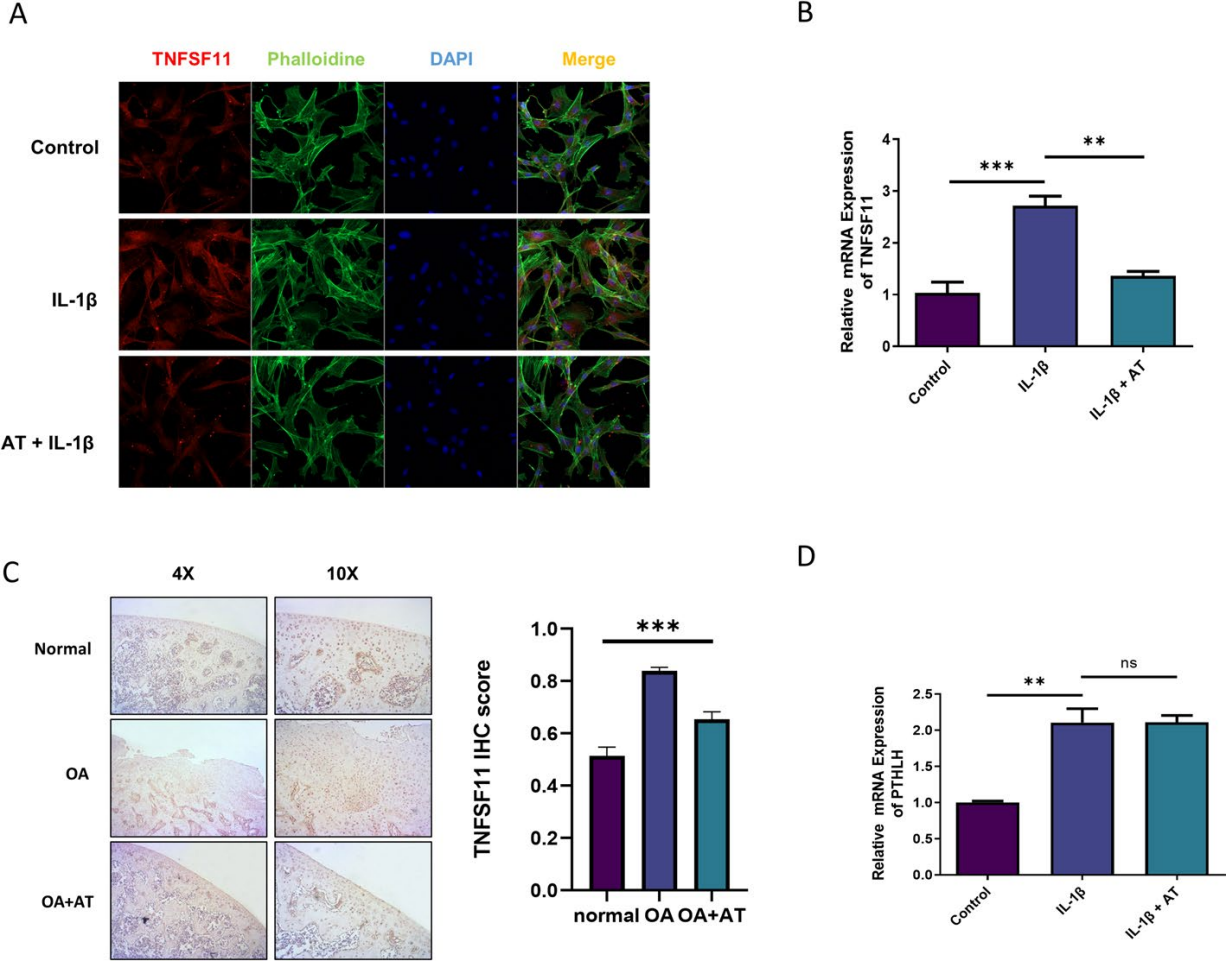
*TNFSF11* was upregulated in OA and downregulated by AT. **A** Immunofluorescence staining for *TNFSF11*, Phalloidin was stained for cellular actin, and DAPI was stained for nuclear. Control: without IL-1β; IL-1β group: with 10 ng/mL; IL-1β + AT group: with 10 ng/mL IL-1β and 20 μM AT. **B** Real-time RT-PCR was performed to determine the gene expression of *TNFSF11*. **C** Immunohistochemistry in OA of the rat was performed to determine the protein level of *TNFSF11*. **D** Real-time RT-PCR was performed to determine the gene expression of PTHLH. All experiments were performed in triplicate. Data represent the mean ± SD (*n* = 3), **P < 0.01, ***P < 0.001

On the basis of the above-mentioned bioinformatic analysis, the expression of *TNFSF11* was significantly downregulated in the IL-1β + AT group. *TNFSF11* gene encodes RANKL protein, which plays a key role in osteoclast differentiation and is crucial to subchondral bone alterations in OA. Further, the OPG–RANK–RANKL pathway is involved in the regulation of knee OA, and the pathways are related to the inflammatory mechanism [34]. Therefore, we first tested the expression of *TNFSF11* in different groups by immunofluorescence staining. Figure 3A shows that the expression of *TNFSF11* in the IL-1β group was higher than that in the control group, and the expression of *TNFSF11* in the IL-1β + AT group was significantly lower than that in the IL-1β group, thereby indicating that *TNFSF11* was upregulated in IL-1β-induced OA and downregulated after AT treatment. qPCR and immunohistochemical analysis were also performed to detect the expression of *TNFSF11*. The results were similar to those obtained via immunofluorescence staining (Fig. 3B, C). PTHLH expression, which plays a vital role in osteoblasts, also significantly changed in the IL-1β + AT group. Therefore, qPCR was used to detect the gene expression. As shown in Fig. 3D, the expression of PTHLH in the IL-1β + AT group was similar to that in the IL-1β group.

### AT alleviated cartilage degradation and defection by activating mitochondrial autophagy through inhibiting PI3K/AKT/mTOR activation in IL-1β-induced chondrocytes

**Fig. 4:**
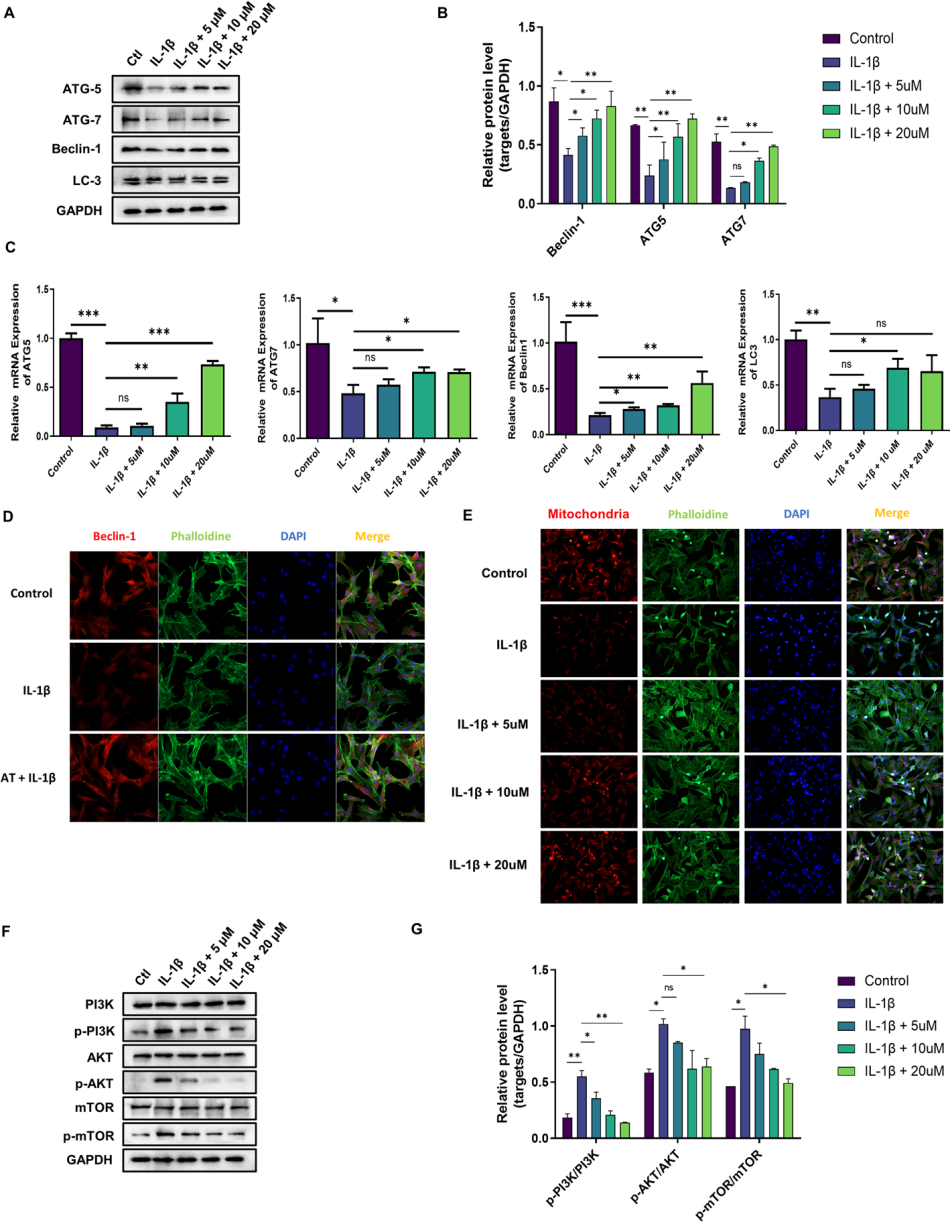
Anti-arthritic effect of AT on IL-1β induced chondrocytes via activating mitochondrial autophagy through inhibiting PI3K/AKT/mTOR. **A** Western blot was used to analyze the proteins ATG-5, ATG-7, beclin-1, and LC-3, which are autophagy-related proteins. **B** Protein level of ATG-5, ATG-7, beclin-1, and LC-3 normalized to GAPDH using ImageJ software. **C** Real-time RT-PCR was performed to determine the gene expression of ATG-5, ATG-7, beclin-1, and LC-3. **D** Immunofluorescence staining for beclin-1, phalloidin was stained for cellular actin, and DAPI was stained for nuclear. Control: without IL-1β; IL-1β group: with 10 ng/mL; IL-1β + AT group: with 10 ng/mL IL-1β and 20 μM AT. **E** Immunofluorescence staining for mitochondria; phalloidin was stained for cellular actin, and DAPI was stained for nuclear. Control: without IL-1β; IL-1β group: with 10 ng/mL; IL-1β + AT group: with 10 ng/mL IL-1β and 5, 10 and 20 μM AT. **F** Western blot was used to analyze the proteins of the PI3K/AKT/mTOR signaling pathway, including PI3K, p-PI3K, AKT, p-AKT, mTOR, and p-mTOR. **G** Protein level of PI3K, p-PI3K, AKT, p-AKT, mTOR, and p-mTOR normalized using ImageJ software. All experiments were performed in triplicate. Data represent the mean ± SD (*n* = 3), * *P* < 0.05, ** *P* < 0.01 and *** *P* < 0.001 versus the IL-1β group

It has previously been reported that the PI3K/AKT/mTOR pathway is involved in OA progression [35, 36]. Our bioinformatic analysis also enriched PI3K/AKT/mTOR pathway, and the PI3K/Akt signaling pathway is another key upstream inhibitor of autophagy [36]. We evaluated the function of AT on autophagy activation in OA chondrocytes. We performed qRT-PCR and western blotting to show that the IL-1β + AT group showed significantly decreased expression of the autophagy-related genes beclin-1, ATG5, ATG7, and LC3 at both mRNA and protein levels when compared with the IL-1β group, and no changes were observed in unstimulated chondrocytes (Fig. 4A, B). AT also significantly increased the conversion of LC3-I to LC3-II, which IL-1β significantly inhibited. Real-time RT-PCR was performed to determine the gene expression of ATG-5, ATG-7, beclin-1, and LC-3, and the results were the same as those of western blotting (Fig. 4C). Immunofluorescence was performed to examine further whether AT could induce autophagy. Beclin-1 significantly increased after AT treatment (Fig. 4D). The mitochondria play a crucial role in the autophagy cascade [37]. Thus, a molecular probe targeted to mitochondria was evaluated to explore the function of AT in mitochondria. As shown in Fig. 4E, mitochondria were activated by AT in a dose-dependent manner compared with the IL-1β group. In conclusion, the above-mentioned findings confirmed that AT reversed IL-1β-induced mitochondrial autophagy inhibition in OA chondrocytes.

On the basis of these results, the involvement of AT in IL-1β-induced cartilage degradation and defection of autophagy by PI3K/AKT/mTOR activation was further investigated. As shown in Fig. 4F, G, IL-1β remarkably increased the phosphorylation levels of PI3K, Akt, and mTOR, whereas AT treatment significantly inhibited IL-1β-associated phosphorylation of substrates in the PI3K/AKT/mTOR pathway, including p-PI3K, p-AKT, and p-mTOR.

**Fig. 5:**
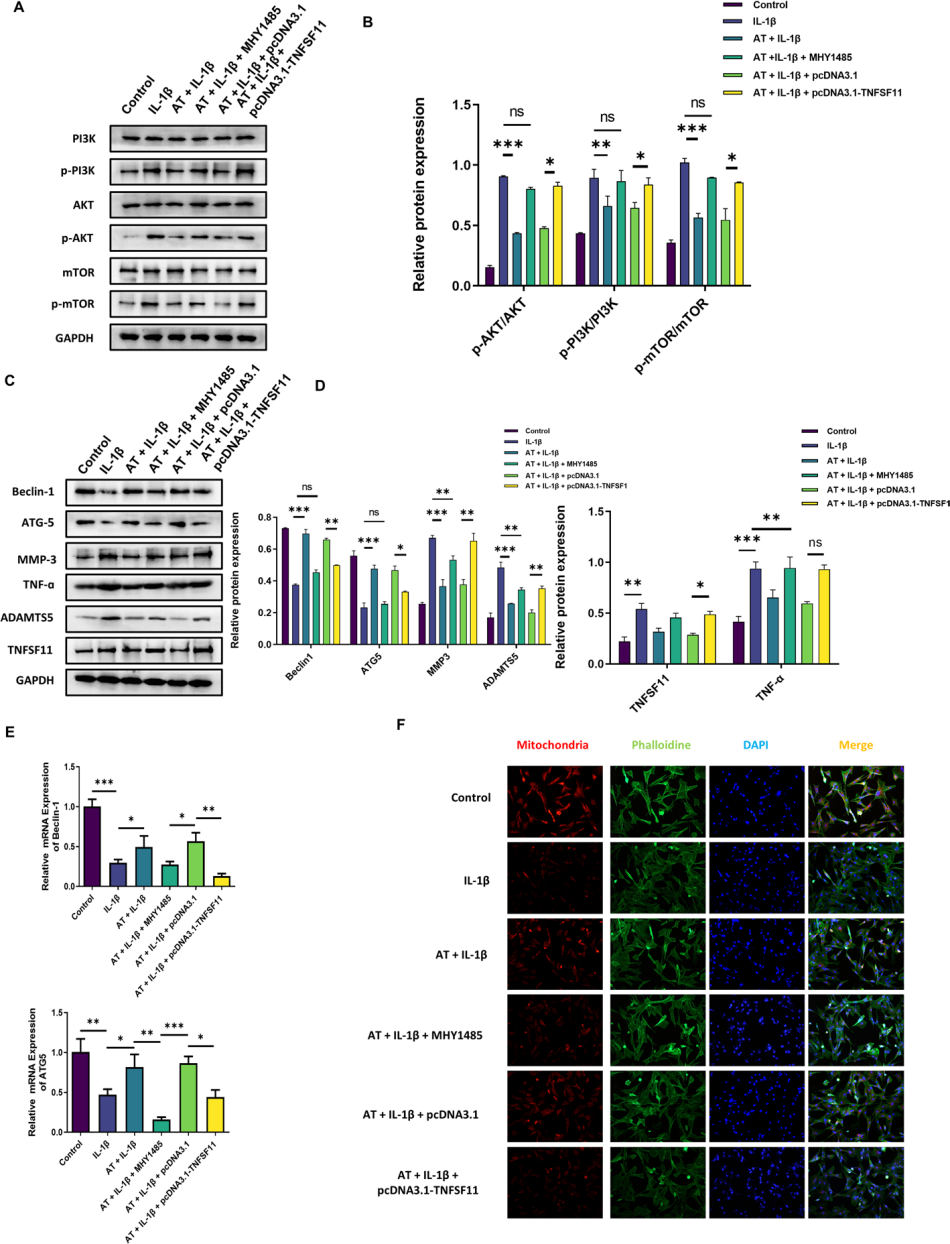

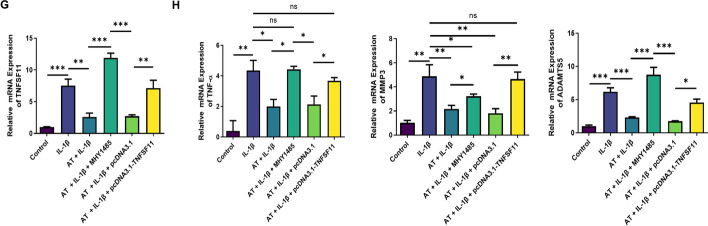
AT relieved osteoarthritis by activating autophagy through reducing *TNFSF11* expression. **A** Western blot was used to analyze the proteins of the PI3K/AKT/mTOR signaling pathway, including PI3K, p-PI3K, AKT, p-AKT, mTOR, and p-mTOR. Three experiments were performed. **B** Protein level of PI3K, p-PI3K, AKT, p-AKT, mTOR, and p-mTOR normalized to GAPDH using ImageJ software. **C** Western blot was used to analyze the proteins of ATG-5, beclin-1, MMP-3, TNF-α, ADAMTS5, and TNFSF11. **D** Proteins levels of ATG-5, beclin-1, MMP-3, TNF-α, ADAMTS5, and TNFSF11 normalized to GAPDH using ImageJ software. **E** Real-time RT-PCR was performed to determine the gene expression of ATG-5 and Beclin-1. **F** Immunofluorescence staining for mitochondria, Phalloidin was stained for cellular actin, and DAPI was stained for nuclear. Control: without IL-1β; IL-1β group: with 10 ng/mL; IL-1β + AT group: with 10 ng/mL IL-1β and 20 μM AT with MHY1485, pcDNA3.1, and pcDNA3.1-*TNFSF11*. **G** Real-time RT-PCR was performed to determine the gene expression of *TNFSF11*. **H** Real-time RT-PCR was performed to determine the gene expression of MMP-3, TNF-α, and ADAMTS5. All experiments were performed in triplicate. Data represent the mean ± SD (*n* = 3), * *P* < 0.05, ** *P* < 0.01 and *** *P* < 0.001 vs. the IL-1β group

MYH1485, an mTOR signaling pathway activator, was used to study the role of the PI3K/AKT/mTOR pathway in OA and further demonstrate AT function. MYH1485 activated the expression of many proteins, including p-PI3K, p-AKT, and p-mTOR, in the PI3K/AKT/mTOR pathway (Fig. 5A, B). Further, treatment with MYH1485 also reversed autophagy activation caused by AT and decreased the expression levels of autophagy-associated beclin-1 proteins and ATG5 (Fig. 5C–E). We also explored whether MHY1485 could reverse the function of AT in terms of mitochondrial autophagy. Figure 5F shows that MHY1485 decreased mitochondrial marker levels, revealing that inhibition of the PI3K/AKT/mTOR signaling pathway reversed the function of AT in mitochondrial autophagy. As shown in Fig. 5C, D, and H, treatment with MYH1485 reversed the protective effect on AT-treated OA chondrocytes, increasing the expression of inflammatory factor TNF-α and significantly increasing the expression of matrix degradation-related proteins, including MMP3, and ADAMTS5. These results indicated that AT treatment enhanced the autophagy ability of OA chondrocytes by regulating the PI3K/Akt/mTOR pathway, the negative functional regulator of autophagy.

### AT relieves osteoarthritis by activating autophagy through reducing *TNFSF11* expression

*TNFSF11* was upregulated in OA and downregulated after AT treatment. *TNFSF11* plays a vital role in IL-1β-induced chondrocytes. To further study the function of *TNFSF11* in AT-induced enhanced autophagy in OA, *TNFSF11* was overexpressed with the help of pcDNA3.1, and blank pcDNA3.1 was the negative control. The efficiency of overexpression was detected via western blot and real-time RT-PCR (Fig. 5C, G). As shown in Fig. 5C, D, and H, overexpression of *TNFSF11* reversed the function of AT, which increased the expression of inflammatory factor TNF-α and autophagy inhibition caused by IL-1β, decreased the expression of autophagy-associated beclin-1 proteins and ATG5, and significantly increased the expression of matrix degradation-related proteins, including MMP3, and ADAMTS5, compared with the control group. Immunofluorescence revealed that the overexpression of *TNFSF11* could reverse the function of AT in mitochondrial autophagy (Fig. 5F). Overexpression of *TNFSF11* can synergistically reverse the protective effect on AT-treated OA chondrocytes. Thus, our result indicated that AT aggravated cartilage degradation and defection by decreasing *TNFSF11* in IL-1β-induced chondrocytes.

### AT alleviated cartilage degradation and the defection of autophagy in rat OA models

**Fig. 6:**
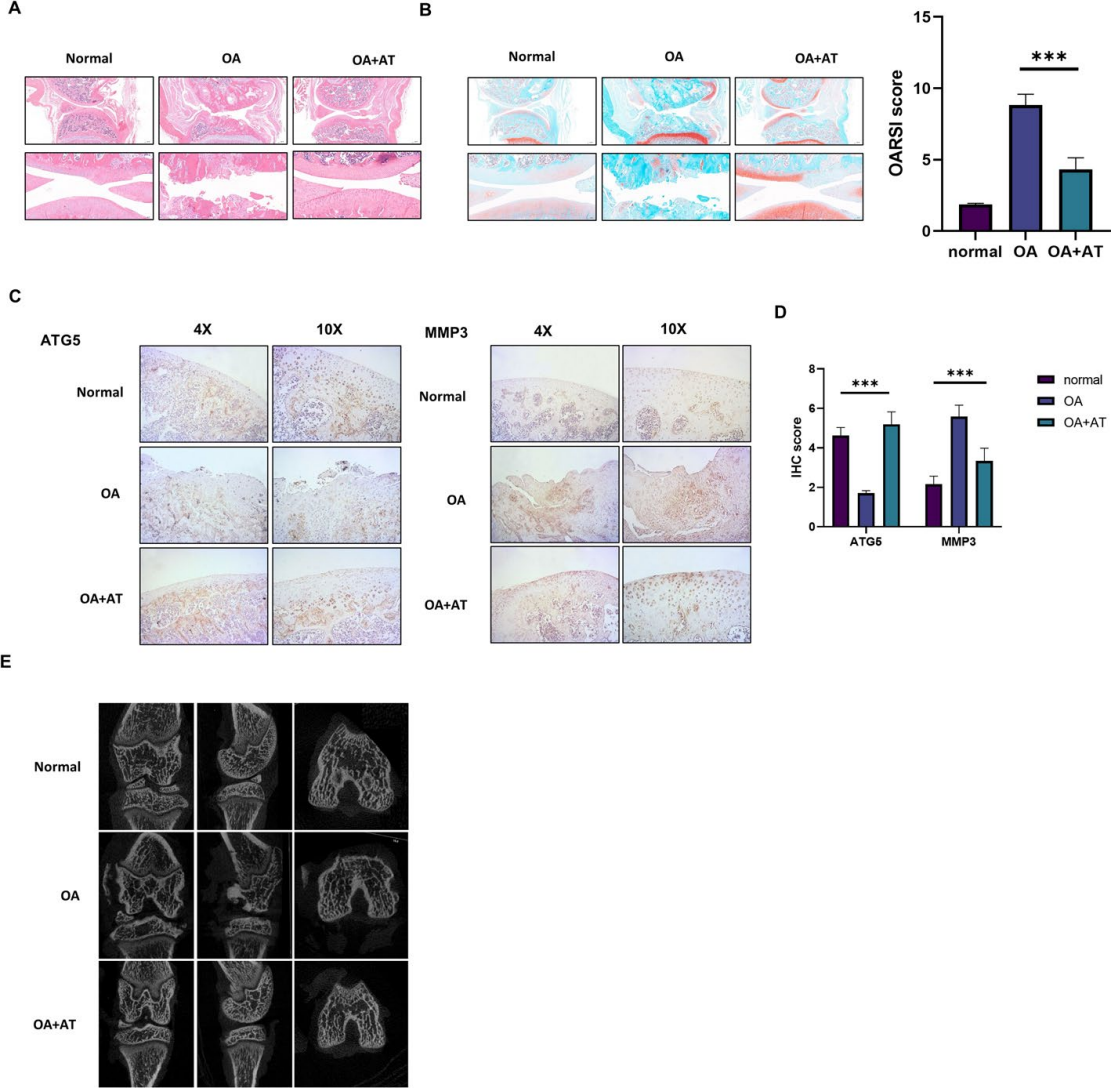
Effect of AT on the treatment of OA in vivo. **A** and **B** Histological sections from knees (*n* = 6) stained with HE and Safranin-O and associated OARSI scores. **C** and **D** Immunohistochemical staining of ATG5. Immunohistochemical staining of MMP3 and quantification of IHC scores. **E** Micro-CT was performed to scan the morphology of the knee joint. Normal group (injected with 0.1 mL PBS, *n* = 5), OA model group (injected with 0.1 mL PBS, *n* = 5), OA + AT group (injected with 0.1 mL of AT, 20 μm, *n* = 5); values are presented as mean ± SD (*n* = 5), *** *P* < 0.001

Given the findings of AT in vitro, a rat OA model was developed to evaluate the corresponding effects in vivo. AT was injected into the knee joints of OA rats and normal rats for 4 weeks. After sacrificing rats using an overdose of anesthesia, knee joint samples were extracted and fixed with 4% paraformaldehyde for 2 days and scanned with micro-computed tomography. The results revealed that treatment with AT attenuated OA (Fig. 6E). Cartilage was collected for histological evaluation. Significantly increased Safranin O staining and minor cartilage destruction were discovered in the OA + AT group compared with that in the OA group (Fig. 6A–C). Immunohistochemical analysis was performed to determine the expression of ATG5, and Fig. 6D revealed that AT treatment activated autophagy. These results suggested that AT treatment significantly attenuated OA in rats.

## Discussion

OA is a chronic degenerative disease characterized by structural, functional, and metabolic changes in all compartments of joints. Joints show progressive articular cartilage degeneration, synovitis, and subchondral bone damage, resulting in physical disability and affecting patients’ quality of life [38, 39]. Currently, most of the treatment methods for OA are symptomatic treatments for pain relief, but no complete cure for OA exists. These methods include nonsteroidal anti-inflammatory drugs, cyclooxygenase 2 (COX-2) inhibitors and hyaluronic acid [6, 40]. Therefore, finding an efficient method to improve cartilage function in OA is urgent.

AT has shown strong anti-inflammatory effects by inhibiting the expression of chemokines and cytokines such as IL-1β, IL-6, and TNF-α. These cytokines are activated by proteases including MMPs, and also act as catabolic factors in cartilage destruction [40, 41]. The disruption of extracellular matrix (ECM) homeostasis plays an important role in OA pathogenesis, and MMP-13 and ADAMTS5 are the most essential regulatory factors because of they can degrade various ECM components [42, 43]. In this study, AT downregulated the expression of pro-inflammatory cytokines in IL-1β-induced chondrocytes and effectively reduced MMP expression, reversing subsequent degradation of the articular cartilage matrix. AT has good biocompatibility and chondroprotective potential and showed increased cell proliferation and viability after IL-1β treatment [44]. The protective effect of AT on cartilage may be related to the prevention of chondrocyte apoptosis. Increasing evidence indicates that chondrocyte apoptosis plays a vital role in the occurrence and development of OA [45, 46]. When apoptosis is activated, AT inhibits the expression of BAX and caspase-3 and the subsequent breakdown of cell components to inhibit cell death [47]. Therefore, reducing the apoptosis rate of chondrocytes during cartilage repair may be an effective cartilage protective mechanism.

We performed RNA sequencing to determine the gene expression in two groups of chondrocytes (IL-1β group and IL-1β + AT group) and found that several mRNAs were related to the PI3K/AKT/mTOR signaling pathway, among which *TNFSF11* was the most significant gene. KEGG and GO analyses confirmed that this DEG was enriched in the PI3K/AKT/mTOR signaling pathway. In IL-1β-induced chondrocytes, the expression of p-PI3K, p-AKT, and p-mTOR was significantly increased, and AT could inhibit the activation of the pathway and decrease the expression of these proteins. Further, MYH1485 is the most commonly used activator of the PI3K/AKT/MTOR pathway [48]. Western blotting showed that, after MYH1485 treatment, autophagy activity was inhibited, matrix degradation was significantly increased, and MYH1485 could reverse AT functions in terms of autophagy activation and matrix degradation protection. These results suggest that the cartilage protection mechanism of AT may be regulated by PI3K/AKT/mTOR signaling in chondrocytes. Real-time RT-PCR and immunofluorescence staining results showed that *TNFSF11* expression significantly increased in OA but significantly decreased after AT treatment. Therefore, *TNFSF11* was considered to be the target gene of AT. To further study the biological function of *TNFSF11* in OA, we constructed a RANKL overexpression plasmid to explore the mechanism of *TNFSF11* in OA. The results suggested that, after overexpression of *TNFSF11*, autophagy activity was inhibited, and matrix degradation was significantly increased, suggesting that chondroprotective and anti-inflammatory effects of AT on IL-1β-induced chondrocytes relied on the downregulation of *TNFSF11* expression. AT has been reported to inhibit osteoclast formation by inhibiting RANKL-induced signaling pathways to alleviate osteoporosis [49, 50]. Our study found that AT alleviated osteoarthritis by downregulating *TNFSF11* expression in chondrocytes. Abnormal subchondral bone remodeling is the main pathological feature of osteoarthritis. The inflammation of articular cartilage in osteoarthritis induced osteoclast formation to absorb the inflammatory bone, but excessive inflammatory activation enhanced bone resorption and destroyed subchondral bone remodeling procession [51]. AT may regulate the multicellular effect, on the one hand reducing osteoclast formation and bone resorption by inhibiting *TNFSF11*-induced signaling pathways, and on the other hand, directly acting on chondrocytes to reduce the inflammatory response and activate autophagy to relieve the symptoms of osteoarthritis.

On the basis of the results of in vitro experiments, we further evaluated this result by injecting AT into the joints of rats to reduce the progression of the disease in vivo. The results suggested that AT could cure OA by activating autophagy. However, some limitations existed in our study. First, we studied the effects of AT in a rat OA model, but the metabolic mechanism of the human body is far more complex than that of a rat, and the effects of AT in vivo cannot be truly simulated for humans. Second, in this study, we focused on the effect of AT on cartilage. However, in vivo, AT cannot only act on chondrocytes. Inevitably, AT will also be produced by other cells or tissues. Therefore, the effect of AT on OA needs further research. For example, AT probes could be designed to specifically target articular cartilage, thereby avoiding the effects of AT on other organ tissues and reducing side effects.

**Fig. 7:**
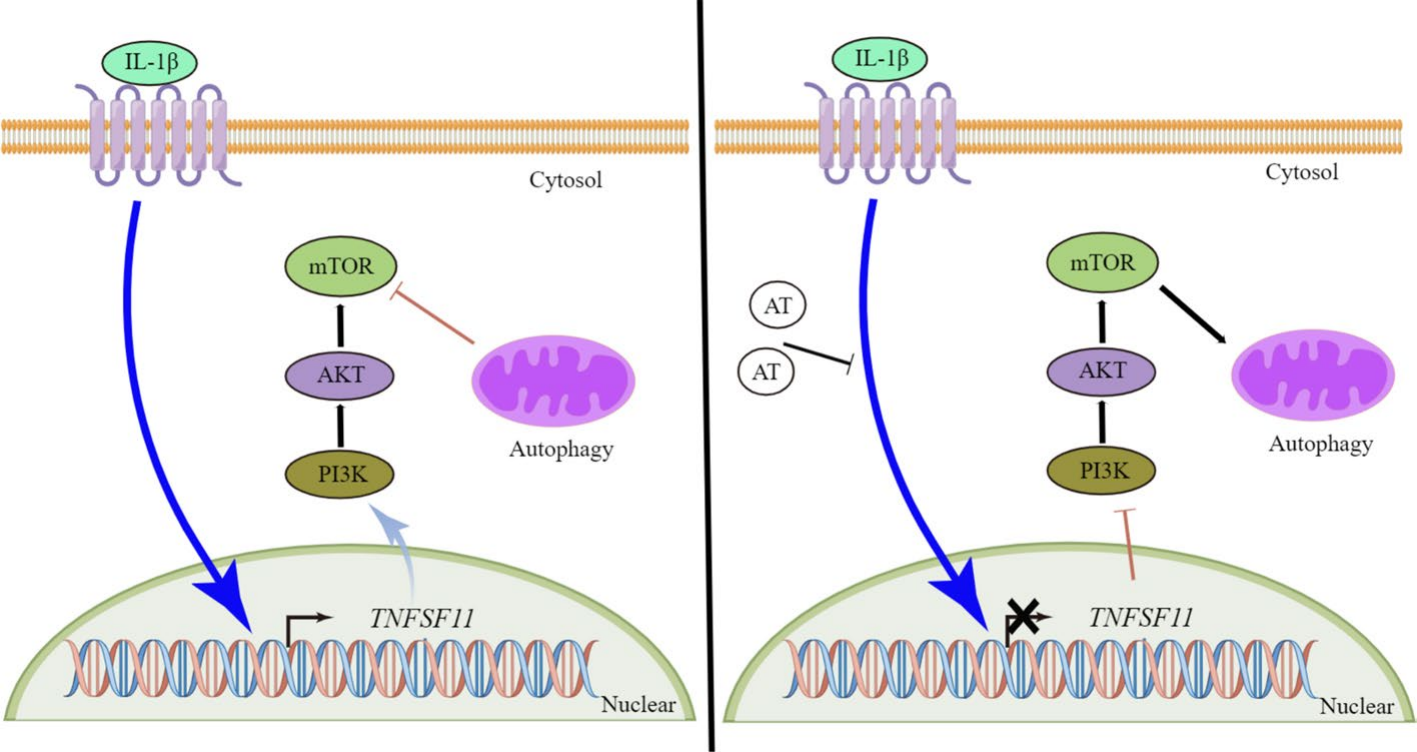
Schematic working model. AT suppressed the expression of *TNFSF11* and inhibited the PI3K–AKT–mTOR pathway in IL-1β induced OA. In the absence of AT, *TNFSF11* activated the PI3K–AKT–mTOR pathway to inhibit autophagy in IL-1β induced OA. In the presence of AT, downregulated *TNFSF11* suppressed the PI3K–AKT–mTOR pathway to promote autophagy in IL-1β-induced OA

In conclusion, the results above suggest that AT has beneficial effects on osteoarthritic chondrocytes and potentially relieves OA by activating autophagy by reducing *TNFSF11* expression and inhibiting PI3K/AKT/mTOR signaling in cartilage, thereby providing novel perspectives and therapeutic strategies for OA (Fig. 7).

## Data Availability

All remaining data are availability within the article, or available from the corresponding authors upon request.
